# Differential expression of decorin, EGFR and cyclin D1 during mammary gland carcinogenesis in TA2 mice with spontaneous breast cancer

**DOI:** 10.1186/1756-9966-29-6

**Published:** 2010-01-22

**Authors:** Yanjun Gu, Shiwu Zhang, Qiang Wu, Shaoyan Xu, Yanfen Cui, Zhengduo Yang, Xiulan Zhao, Baocun Sun

**Affiliations:** 1 Department of Pathology, Tianjin Cancer Hospital and Institute, Tianjin Medical University, Tianjin 300060, China; 2 Department of Pathology, Medical College of the Chinese People's Armed Police Force, Tianjin 300162, China; 3 Department of Pathology, Anhui Medical University, Meishan Road 81#, Shushan District, Hefei, Anhui province, 230032, China; 4 Department of Pathology, Tianjin Medical University, Tianjin 300060, China

## Abstract

**Background:**

The Tientsin Albino 2 (TA2) mouse is an inbred strain originating from the Kunming strain. It has a high incidence of spontaneous breast cancer without the need for external inducers or carcinogens. Until now, the mechanism of carcinogenesis has remained unclear. In this study, we investigate differential gene expression, especially the expression of decorin, EGFR and cyclin D1, during mammary gland epithelial cell carcinogenesis in TA2 mice.

**Methods:**

Gene expression profiles of spontaneous breast cancer and matched normal mammary gland tissues in TA2 mice were ascertained using an Affymetrix Mouse 430 2.0 array. Twelve mammary tissue samples from five month-old female TA2 mice (Group A), as well as 28 samples from mammary (Group B) and cancer tissues (Group C) of spontaneous breast cancer-bearing TA2 mice, were subsequently used to detect the expression of decorin, EGFR and cyclin D1 by real-time PCR and immunohistochemical methods.

**Results:**

Several imprinted genes, oncogenes and tumor suppressor genes were differentially expressed between normal mammary gland tissues and breast cancer tissues of TA2 mice. The imprinted gene decorin and the oncogene EGFR were down-regulated in tumor tissues, while the oncogene cyclin D1 was up-regulated. Immunohistochemistry showed that samples in Group A showed high decorin expression more frequently than those in Group B (*P *< 0.05). More tissue samples in Group B than Group A were positive for nuclear EGFR, and tissue samples in Group B more frequently showed high nuclear EGFR expression than those in Group A or Group C (*P *< 0.05). The labeling index for cyclin D1 in Group C was significantly higher than in Group B. Mammary tissues of Group A expressed the highest level of decorin mRNA (*P *< 0.05), and mammary tissues of Group B expressed the highest level of EGFR mRNA (*P *< 0.05), while cancer tissues expressed the highest level of cyclin D1 mRNA (*P *< 0.05).

**Conclusions:**

The expression of decorin, EGFR and cyclin D1 in mammary epithelial cells changes with increasing age. The abnormal expression of them may partly contribute to the genesis of spontaneous breast cancer in TA2 mice.

## Background

The Tientsin Albino 2 (TA2) mouse is an inbred strain originating from the Kunming strain. It has a high incidence of spontaneous breast cancer without the need for external inducers or carcinogens. The morbidity in parous females is 84.1% within an average of 280 days after birthing a litter [1-3]. Until now, the mechanism of carcinogenesis has remained unclear. Gene expression arrays are commonly used in cancer research to identify differentially expressed candidate genes under two different conditions [4,5]. The Affymetrix expression array is one of the most widely used commercially available oligonucleotide arrays and can determine the gene expression status of virtually the complete genome at the mRNA level.

Genomic imprinting is an epigenetic process that marks the parental origin of a subset of genes, resulting in the silencing of specific alleles [6]. To date, more than 70 imprinted genes have been described in the mouse http://www.mgu.har.mrc.ac.uk/imprinting/imprinting.html. Proteins encoded by these genes include growth factors and components of the extracellular matrix (ECM). Imprinted genes are involved in several cellular processes and perform a variety of functions, including cell cycle control, G-protein-coupled receptor signaling, and intracellular signaling, thereby influencing both pre- and postnatal growth and development through endocrine/paracrine pathways[6]. More recent data have shown that abnormal expression of several imprinted genes including decorin can cause tumorigenesis. Decorin is a maternally expressed imprinted gene that belongs to the small leucine-rich proteoglycan (SLRP) gene family and has been implicated in the control of cell proliferation [7,8]. Reduced expression of decorin facilitates tumorigenesis and cell growth [9,10]. Decorin is a functional component of the ECM, and is also considered to be a novel biological ligand for EGFR, which is frequently expressed at elevated levels in multiple cancers of epithelial origin. Interactions between these factors can inhibit cell growth during tissue remodeling and cancer development [11]. In addition to serving as a ligand for EGFR, decorin can bind to various forms of active TGF-β through its core protein and can neutralize the activity of TGF-β[12]. Abnormal expression of decorin has been found in many tumors, including lymphoma and human breast carcinoma [13,14].

In this study, gene expression profiles of normal mammary glands and spontaneous breast cancer tissues from TA2 mice were detected by Affymetrix Mouse Genome 430 2.0 Arrays for the first time. The expression data were analyzed by the MAS5.0 [4], BGX[15], and Array2BIO[16] methods. Based on the candidate genes identified by expression profiling, we hypothesized that abnormal expression of decorin, EGFR, and cyclin D1 might induce carcinogenesis of mammary gland epithelial cells in TA2 mice.

## Methods

### Animals and Sampling

Female TA2 mice (five month-old TA2 mice and spontaneous breast cancer-bearing TA2 mice) were purchased from the Experimental Animal Center of Tianjin Medical University. The Animal Ethics Committee of National Research Institute for Family Planning Beijing approved the animal experimentation protocols and all animal experiments were performed according to guidelines (Guidelines for the Care and Use of Laboratory Animals) established by the Chinese Council on Animal Care.

A total of 12 five month-old mice and 28 cancer-bearing mice were used in this study. As for the 28 cancer-bearing mice, spontaneous breast cancer was found with an average of 307 days after birth (213 days to 408 days). After euthanasia, mammary glands and spontaneous breast cancer tissues were collected from each cancer-bearing animal. Two abdominal mammary glands were collected from the five month-old mice (Group A). One was immediately frozen in liquid nitrogen and stored at -70°C, and the other was fixed in 4% formalin and embedded in paraffin. For cancer-bearing mice, spontaneous breast cancer tissues (Group C) and normal mammary glands located on the side opposite to the tumor (Group B) were collected. Portions of the tumor tissues and one of the normal mammary glands were immediately frozen in liquid nitrogen and stored at -70°C; the remaining tumor tissues and mammary glands were routinely fixed in 4% formalin and embedded in paraffin.

### RNA Extraction

Total RNA was extracted from frozen tissue using Trizol reagent (Invitrogen, Paisley, UK) and isolated using the RNeasy extraction protocol from Qiagen (Valencia, CA, USA). The integrity of total RNA for each sample was determined by denaturing gel electrophoresis (1.2% methyl aldehyde running gel), and the purity of RNA was checked by spectrophotometry. The O.D. 260/280 nm ratio was between 2.05-2.15 for each RNA sample.

### Microarray hybridization and data analysis

Total RNA from six samples harvested from three mice (three samples each for Groups B and C) was used for microarray hybridization. Microarray analysis was conducted using Affymetrix (Santa Clara, CA) Mouse Genome 430 2.0 Arrays (over 39,000 transcripts and variants from over 34,000 well characterized mouse genes). The procedure was conducted according to the manufacturer's instructions (Affymetrix) using T7-(dT)24-oligonucleotide primers for cDNA synthesis (Affymetrix), the cDNA Cleanup Module (Affymetrix) for purification and the IVT Labeling Kit (Affymetrix) for making biotin-labeled cRNA. Clean-up of cRNA using RNeasy columns (Qiagen, Crawley, Sussex, UK) was performed to remove unincorporated ribonucleotides prior to quantification by spectrophotometry. The cRNA was fragmented by metal-induced hydrolysis at 94°C for 35 min in a 40 mM final concentration Tris buffer. The length of the fragmented cRNA was between 25 bp and 200 bp. Adequacy of cRNA fragmentation was determined by 1.2% denaturing gel electrophoresis, and a hybridization control was prepared in hybridization buffer. The Affymetrix GeneChip system was used for hybridization, staining, and imaging of the arrays according to the standard Affymetrix protocol. Hybridization cocktails were hybridized to Mouse Genome 430 2.0 Arrays, after which the arrays were washed using a Fluidics Station 450 (FS450, Affymetrix) and then scanned using a Scanner 3000 7G 4C (Affymetrix). Microarray Suite 5.0 software (MAS5.0, Affymetrix) was used to process images and estimate transcript expression levels. The expression data were analyzed by MAS5.0, BGX and Arrary2BIO methods.

### Real-time PCR

The relative expression levels of decorin, EGFR, cyclin D1 and PCNA were determined by quantitative PCR using SYBR Premix Ex Taq(TaKaRa Code: DRR041A) purchased from TaKaRa Biotechnology (Dalian) Co., Ltd with β-actin as a reference (TaKaRa Code: D3751). Samples were run in separate tubes on an ABI Prism 7500 Sequence Detection System according to the manufacturer's suggested protocols. In brief, the 50 μl samples were treated at 50°C for 2 min and 95°C for 10 s followed by 40 cycles of 95°C for 5 s and 64.2°C (for EGFR and PCNA), 65.0°C (for cyclin D1), or 60°C (for decorin) for 33 s. Relative quantitation using the comparative CT method was performed for each sample. Primers were synthesized by TaKaRa Biotechnology (Dalian) Co., Ltd. with the following sequences: decorin (GenBank accession no. NM_007833), forward 5'-TGATGCACCCAGCCTGAAAG-3', reverse 5'-TCCATAACGGTGATGCTGTTGAA-3'; EGFR (GenBank accession no. NM_207655), forward 5'-AGGACTGGGCAATCTGTTGGA-3', reverse 5'-GAAGATCGAAGACCTGGTGCTGTAA-3'; PCNA (GenBank accession no. NM_011045), forward5'-GGACTTAGATGTGGAGCAACTTGGA-3'; reverse 5'-AATTCACCCGACGGCATCTTTA-3'; cyclin D1 (GenBank accession no. NM_007631), forward 5'-AGTCAGGGCACCTGGATTGTTC-3', reverse 5'-AACAGATTAAATGATGCACCGGAGA-3'. Experiments were performed in triplicate for each sample.

### Immunohistochemistry

Formalin-fixed and paraffin-embedded mammary gland and spontaneous breast cancer specimens were used for immunohistochemical detection of decorin, EGFR, cyclin D1 and PCNA. Sections 4 μm in thickness were deparaffinized and rehydrated with xylene and graded alcohol solutions. After washing with PBS, endogenous peroxidase activity was quenched by 3% hydrogen peroxide, and sections were boiled in 10 mM citrate buffer (pH 6.0) for 3 min in an autoclave sterilizer followed by cooling at room temperature for more than 20 min. After rinsing with PBS, sections were incubated with primary antibodies (1:100 dilution in antibody diluent, Zhongshan Goldbridge Biotechnology CO., Ltd, Beijing, China) for 18 hr at 4°C. Sections were stained with anti-decorin (SC-73896, Santa Cruz Biotechnology, Inc), anti-EGFR (BA0843, Boster Biological Technology, Ltd, Wuhan, China), anti-cyclin D1 (Cat. #RM-9104-S1, Neomarker Labvision, USA), or anti-PCNA (BM0104, Boster Biological Technology, Ltd, Wuhan, China) antibodies. After rinsing with PBS, sections were incubated with PV6001 or PV6002 (Zhongshan Goldbridge Biotechnology CO., Ltd, Beijing, China) for 30 min at 37°C and stained with DAB (AR1022, Boster Biological Technology, Ltd, Wuhan, China) for 1 to 2 min. The slides were counterstained with hematoxylin, dehydrated with ethanol, cleared with xylene, and mounted in neutral gum. Control sections were incubated with PBS instead of a primary antibody. All slides were analyzed by two independent observers.

### Immunohistochemical staining evaluation

For cyclin D1 and PCNA, only the percentage of immunoreactive epithelial cells and breast cancer cells was considered (labeling index). Briefly, the areas of high percentage of cyclin D1 positive cells ('hot spots') were identified at low magnification (×10 ocular and ×10 objective) as the "hot spots". Then, ten hot spot areas per section were selected and were observed at a higher magnification (×10 ocular and ×40 objective, high power field) with a grid (OLYMPUS 100×) in the ocular lens. All epithelial cells or cancer cells and immunohistochemistry positive cells in the grid were counted in every high power field, respectively. The mean percentage of positive cells (labeling index) was used to evaluate the expression of the protein in a section. For EGFR, both the percentage and intensity of EGFR-positive epithelial cells and breast cancer cells were considered in a semi-quantitative assessment [17]. The percentage of EGFR-positive cells was scored as 0 (0% positive cells), 1 (1-25% positive cells), 2 (26-50% positive cells), 3 (50-75% positive cells), or 4 (>75% positive cells). The intensity of EGFR immunostaining was also scored as 0 (negative), 1 (weak), 2 (intermediate) and 3 (strong). The intensity score (0-3) was multiplied by the percentage score (0-4) and a final score was assigned 0 (negative), 1-4 (weak expression), 5-8 (moderate expression), and 8-12 (strong expression). Samples with scores of 0-4 were considered to show low expression, while those with scores of 5-12 were considered to show high expression. For decorin, the percentage of decorin-positive cells or decorin-positive areas located around the terminal duct and gland alveolus was scored as 0 (0% positive cells or substance), 1 (1-25% terminal duct and gland alveolus), 2 (26-50% terminal duct and gland alveolus), or 3 (>50% terminal duct and gland alveolus), and samples with scores of 3 were considered to show high expression. In tumor tissues, the distribution of decorin-positive cells or decorin-positive areas was recorded.

### Statistical Analysis

All data were analyzed using SPSS statistical software (version 11.5 for Windows). The Kruskal-Wallis and Mann-Whitney tests were used to evaluate statistical significance of differences, and the Spearman rank test was used to assess the correlation between the expression of EGFR and cyclin D1 or PCNA. Differences were considered statistically significant at *P *< 0.05.

## Results

### **Differentially expressed imprinted genes and oncogenes between normal mammary glands and spontaneous breast cancer tissues**

Expression profiles of spontaneous breast cancer and matched normal mammary glands were obtained using the Affymetrix GeneChip Mouse430 2.0 oligonucleotide array. In total, 260 differentially expressed candidate genes (data not shown) were detected by all three analysis methods (MAS5.0, BGX, Array2BIO). These genes included five imprinted genes and seven oncogenes or tumor suppressor genes (Table 1). Of these genes, the imprinted gene decorin and the oncogene EGFR were down-regulated in tumor tissues as compared to normal mammary gland tissues, and the oncogene cyclin D1 was up-regulated in tumor tissues.

**Table 1:** Differentially expressed candidate imprinted genes, oncogenes and tumor suppressing genes identified by MAS5.0, Array2BIO and BGX methods

Gene symbol	Description	Genbank	Biological process	Biological function
**Imprinted gene**				
Dcn	Decorin	AV321547	----	protein binding
Igf2	Insulin like growth factor 2	NM010514	regulation of cell cycle; cell proliferation	receptor binding; hormone activity; protein binding; growth factor activity
Mest	Mesoderm specific transcript	AW555393	Proteolysis	Catalytic activity; aminopeptidase activity
Ndn	Necdin	BB074430	Regulation of cell growth; nerve growth factor receptor signaling pathway; transcription; neuron migration	DNA binding; protein binding; gamma-tubulin binding
Peg3	Paternally expressed 3	BB305002	apoptosis	Nucleic acid binding; metal ion binding
**Oncogenes or tumor suppressor genes**				
Bcl11a	B-cell CLL/lymphoma 11A	BF731393	transcription; B cell and T cell differentiation	nucleic acid binding; zinc ion binding; transcription co-repressor activity
Ccnd1	cyclin D1	BB452046	Regulation of cell cycle; protein amino acid phosphorylation; fat cell differentiation; cell division	protein kinase activity; protein binding
Cdh11	Cadherin 11	BG072720	Cell adhesion	Calcium ion binding; protein binding
Egfr	Epidermal growth factor receptor	BB409522	Cell morphogenesis; signal transduction; cell proliferation; regulation of cell migration; cell adhesion; regulation of peptidyl-tyrosine phosphorylation	Nucleotide binding; kinase activity; iron ion binding; ATP binding; protein binding; signal transducer activity; receptor activity; transferase activity; actin filament binding
Fcgr2b	Fc receptor, IgG, low affinity IIb	BB530063	Negative regulation of type I hypersensitivity; immune response; signal transduction; regulation of B cell proliferation; mast cell activation; regulation of phagocytosis	Receptor activity; protein binding; IgG binding
Gpc3	Gypican 3	BM198842	Ureteric bud branching; regulation of cell proliferation; regulation of BMP signaling pathway; regulation of growth	GPI anchor binding
Vav3	Vav 3 oncogene	BM224051	Vesicle fusion; signal transduction; regulation of cell adhesion; cell migration; intracellular signaling cascade; cell projection biogenesis; regulation of PI3 kinase activity; integrin-mediated signaling pathway	diacylglycerol binding; EGFR binding; metal ion binding; exchange factor activity

### Expression of decorin in normal mammary glands and spontaneous breast cancer tissues

**Figure 1:**
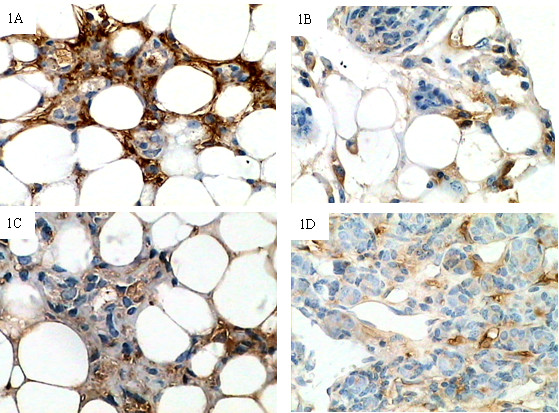
**Expression of decorin in mammary glands and spontaneous breast cancer tissues from TA2 mice**. 1A, 1B, Decorin-positive structures were located around the terminal duct and gland alveolus in five-month-old TA2 mice and was mainly expressed by mesenchymal cells (IHC, 200×). 1C, Decorin-positive structures were located around the terminal duct and gland alveolus from tumor-bearing TA2 mice (IHC, 200×). The mammary glands of young mice expressed higher levels of decorin than those of spontaneous cancer-bearing mice. 1D, Decorin-positive structures were present in the ECM of tumor tissues (IHC, 200×).

To localize decorin protein, immunohistochemical analysis was performed using normal mammary gland and spontaneous breast cancer samples. Decorin was expressed in normal mammary gland tissues of five month-old TA2 mice (Group A) and breast cancer-bearing TA2 mice (Group B), as well as in breast cancer tissues from TA2 mice (Group C). It was mainly present in the ECM and expressed by mesenchymal cells, such as fibroblasts and some inflammatory cells (Fig 1A). In normal mammary gland tissue samples (Group B), weak decorin staining was present in the ECM, with stronger staining around the terminal duct and gland alveolus, while the epithelial cells were devoid of decorin staining. In contrast, tissues of Group A showed strong decorin staining in the ECM. The strongest decorin-positive structures were located around the terminal duct and gland alveolus (Fig 1B, C). Group A showed a significantly higher percentage of cells highly expressing decorin (high expression in 50%) than Group B (high expression in 17.86%). Tissues in Group B showed a significant decrease in the occurrence of high decorin protein expression (high expression in 17.86%) compared to Group A (high expression in 50%) (χ^2 ^= 4.35;*P *= 0.037). This finding suggests that the mammary glands of young mice expressed higher levels of decorin than those of spontaneous cancer-bearing mice. In Group C, tumor cells exhibited no decorin immunoreactivity, and decorin was only expressed by some mesenchymal cells, with the strongest staining observed in the ECM at the border of the tumor (Fig 1D).

Real-time PCR was performed to evaluate the expression level of decorin mRNA in mammary gland tissues and tumor tissue samples. Normal mammary glands (Group A) expressed the highest level of decorin mRNA among the three groups, and tumor tissues (Group C) expressed the lowest level (Table 2).

**Table 2:** Expression levels of decorin, EGFR, cyclin D1 and PCNA mRNA in mammary glands and spontaneous breast cancer tissues of TA2 mice

Group	Decorin	EGFR	Cyclin D1	PCNA
Group A	0.95 ± 0.25	0.02 ± 0.01	0.04 ± 0.01	0.14 ± 0.10
Group B	0.27 ± 0.20*	0.05 ± 0.02*	0.13 ± 0.08*	0.38 ± 0.24*
Group C	0.13 ± 0.10^#^	0.03 ± 0.01^#^	0.42 ± 0.22^#^	0.17 ± 0.10^#^

*: compared with Group A, *P *< 0.05; ^#^: compared with Group B, *P *< 0.05

Group A: normal mammary glands from five-month-old TA2 mice; Group B: normal mammary glands from spontaneous breast cancer-bearing TA2 mice; Group C: spontaneous breast cancer tissue from TA2 mice.

### Expression of EGFR in normal mammary glands and spontaneous breast cancer tissues

**Figure 2:**
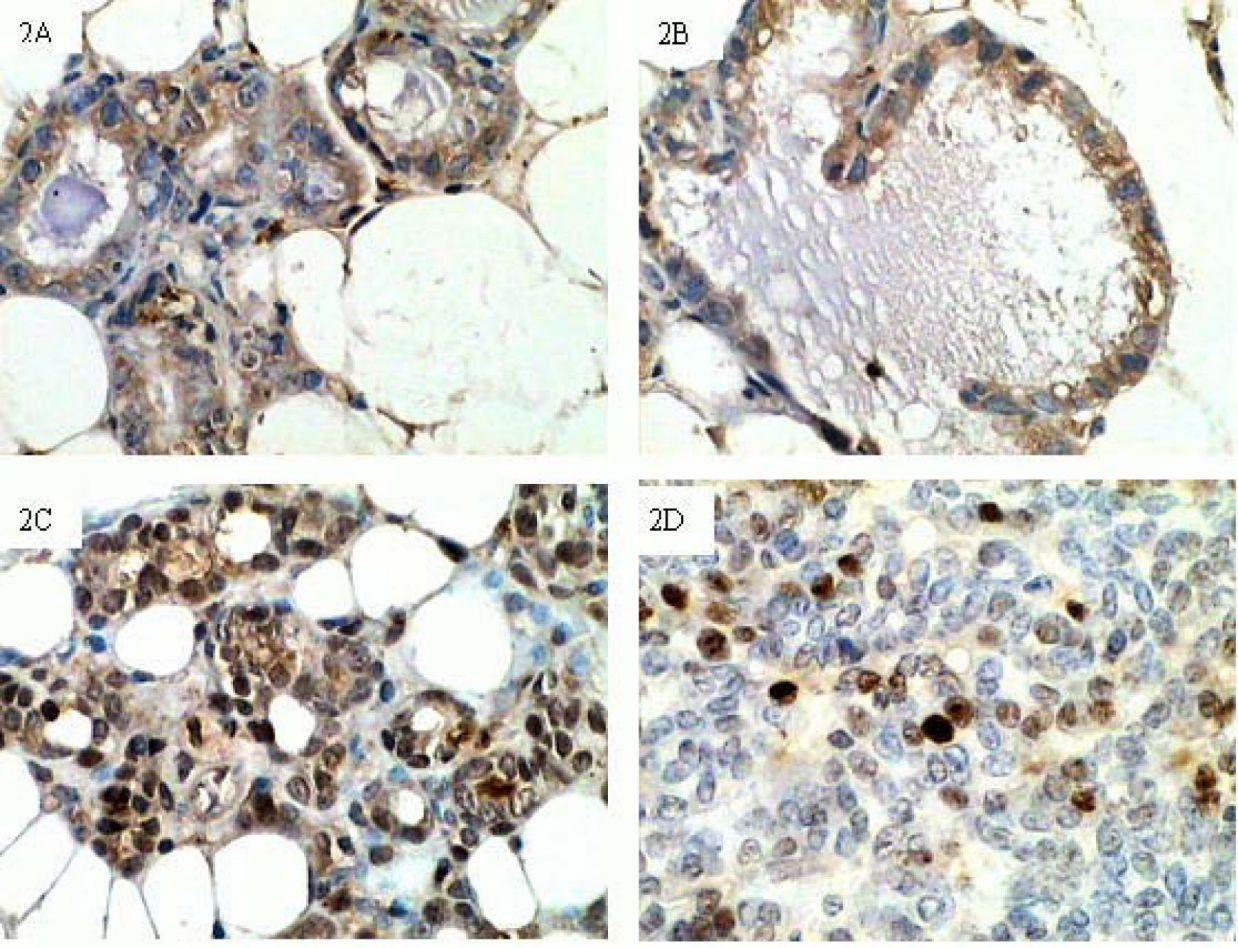
**Expression of EGFR in mammary glands and spontaneous breast cancer tissues from TA2 mice**. 2A, EGFR staining could be observed occasionally in epithelial cells in mammary gland tissues from five-month-old TA2 mice (IHC, 200×). 2B and 2C, EGFR staining was localized to both the cytoplasm and nucleus in mammary gland tissues from spontaneous breast cancer-bearing TA2 mice (IHC, 200×). 2D, Nuclear EGFR was also present in spontaneous breast cancer tissues from TA2 mice (IHC, 200×). Mammary gland tissues and tumor tissues from cancer-bearing TA2 mice expressed higher levels of EGFR than those of mammary gland tissues of five-month-old TA2 mice.

EGFR was expressed by terminal duct epithelial cells, gland alveolus cells and tumor cells, as well as some mesenchymal cells. In Group A, EGFR was mainly expressed by epithelial cells and localized to the cytoplasm (Fig 2A). In spontaneous breast cancer-bearing mice, stronger EGFR staining was observed in mammary gland samples when compared to tumor samples, and nuclear translocation was observed in both tissue types (Fig 2B, C, D). EGFR-expressing samples and EGFR nuclear translocation were also more often observed in Group B than in Group A (respectively: χ^2 ^= 7.56, *P *< 0.01; χ^2 ^= 20.49, *P *< 0.01). High levels of EGFR staining were more often observed in Group B than in Group C (χ^2 ^= 4.14; *P *< 0.05, Table 3); this pattern was supported by real-time PCR data. Normal mammary glands (Group A) showed the lowest level of EGFR mRNA expression, while mammary glands from tumor-bearing mice (Group B) showed the highest expression level (Table 2).

**Table 3:** EGFR staining in normal mammary glands and tumor tissues from TA2 mice(expressed as a percentage of samples with positive staining)

	n	Positive expression	Nuclear translocation	High expression level
Group A	12	33.33(4/12)	0.00(0/12)	0.00(0/12)
Group B	28	78.57(22/28)^#^	53.57(15/28)^#^	42.86(12/28)*
Group C	28	64.29(18/28)^#^	39.28(11/28)^#^	17.86(5/28)

^#^: compared with Group A, *P *< 0.05; *: compared with Group C, *P *< 0.05

Group A: normal mammary glands from five month-old TA2 mice; Group B: normal mammary glands from spontaneous breast cancer-bearing TA2 mice; Group C: spontaneous breast cancer tissue from TA2 mice.

### Expression of cyclin D1 and PCNA in normal mammary glands and spontaneous breast cancer tissues

**Figure 3:**
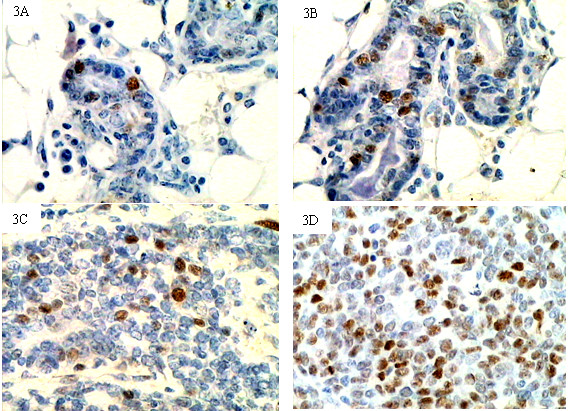
**Expression of cyclin D1 in mammary glands and spontaneous breast cancer tissues from TA2 mice**. 3A, Cyclin D1 staining could be observed occasionally in epithelial cells from five month-old TA2 mice (IHC, 200×). 3B, Cyclin D1 staining was present in the nuclei of epithelial cells in mammary gland tissues of spontaneous breast cancer-bearing TA2 mice (IHC, 200×). 3C, Cyclin D1 staining was present in the nuclei of hyperplastic epithelial cells of spontaneous breast cancer-bearing TA2 mice (IHC, 200×). 3D, Cyclin D1 staining was also present in spontaneous breast cancer tissues of TA2 mice (IHC, 200×). The Labeling Index of cyclin D1 increased apparently from Group A to Group C.

**Figure 4:**
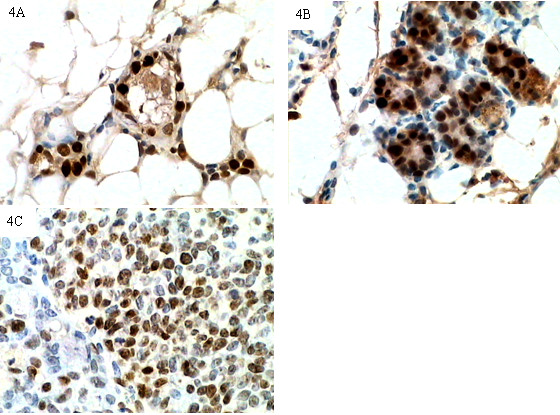
**Expression of PCNA in mammary glands and spontaneous breast cancer tissues from TA2 mice**. PCNA staining could be observed in the nuclei of epithelial cells from five month-old TA2 mice (4A) and spontaneous breast cancer-bearing TA2 mice (4B) (IHC, 400×). PCNA staining was present in the nuclei of spontaneous breast cancer cells from TA2 mice (4C) (IHC, 400×).

Cyclin D1 and PCNA were expressed by terminal duct epithelial cells, gland alveolus cells and tumor cells (Fig 3A-3D, Fig 4A-4C). Some mesenchymal cells also showed cyclin D1 and PCNA staining. In five month-old mice, cyclin D1 staining was observed occasionally in anestric epithelial cells. In mammary gland tissue samples of tumor-bearing mice, most epithelial cells were negative for cyclin D1 staining and several "hot spots areas" (areas with high expression of cyclin D1) were observed. In general, one hot spot area limited to one "mammary gland lobula" which contained several closely distributed terminal duct and gland alveolus. In hot spot areas, the cyclin D1 labeling index in Group C was higher than in Group B (22.33 ± 17.25 vs. 12.25 ± 7.19, Z = -2.25, *P *< 0.05). In Groups B and C, the cyclin D1 labeling index was higher in samples with nuclear EGFR expression than in samples without nuclear EGFR expression (Z = -2.28, *P *< 0.05, Group B; Z = -2.07, *P *< 0.05, Group C, respectively); results are shown in Table 4. Most of the "hot spot" cyclin D1 areas also demonstrate a "hot spot" of nuclear localized EGFR. A positive correlation was found between the cyclin D1 labeling index and the expression level of nuclear EGFR in Groups B and C (*r*_s _= 0.723, 0.474, *P *< 0.05), but no correlation was established between nuclear EGFR expression and the PCNA labeling index. These results suggest that nuclear EGFR could be an upstream effector of cyclin D1 expression. Furthermore, we analyzed the expression of cyclin D1 and PCNA mRNA (Table 3), and found that cyclin D1 mRNA was progressively upregulated in the following relative magnitudes: Group A < Group B < Group C. Because the mammary gland tissues used for immunohistochemical staining and real-time PCR were independent samples, we could not correlate the expression of nuclear EGFR and the expression levels of cyclin D1 mRNA. However, a trend (tendency) of positive correlation was established between the expression level of nuclear EGFR and the expression level of cyclin D1 mRNA for tumor tissue samples that did not reach significance (*r*_*s *_= 0.883, *P *= 0.059). These findings also suggest that nuclear EGFR might partly regulate the expression of cyclin D1.

**Table 4:** Cyclin D1 and PCNA labeling index of normal mammary glands and cancer tissues from spontaneous breast cancer -bearing TA2 mice (%)

	n	Cyclin D1	PCNA
Group B			
Nucleus EGFR (+)	15	15.15 ± 5.16*	37.81 ± 12.77
Nucleus EGFR (-)	13	8.77 ± 7.95	33.71 ± 15.78
Group C			
Nucleus EGFR (+)	11	31.17 ± 12.50*	44.9212.01
Nucleus EGFR (-)	17	18.54 ± 17.98	33.9413.92

*:compared to samples without nuclear EGFR expression, *P *< 0.05

Group B: normal mammary glands from spontaneous breast cancer-bearing TA2 mice; Group C: spontaneous breast cancer tissue from TA2 mice.

## Discussion

Breast cancer is one of the most common malignant tumors in adult females and develops as a result of altered expression of multiple genes and abnormal cellular pathways. In recent years, accumulating data has shown that alterations of the stromal compartment can also influence tumor cell behavior through paracrine growth factor pathways[9]. Proteoglycans are the main constituents of the ECM, and their synthesis and degradation are regulated by many effectors that control the development and function of the mammary gland. We hypothesized that alterations in the composition and architecture of the ECM could promote carcinogenesis of breast epithelial cells. To test this hypothesis, we used tissue samples taken from TA2 mice. Gene expression arrays revealed that several imprinted genes, oncogenes and tumor suppressor genes were differentially expressed between normal mammary glands and spontaneous breast cancer tissues. Some of these genes encoded stromal constituents such as versican and decorin. Decorin is synthesized by the majority of mesenchymal cells [18]. However, it also interacts with a variety of other ECM components and can affect cell growth. It has been shown that decorin functionally inactivates the ErbB2 protein in breast carcinoma cells [18], leading to growth suppression and cytodifferentiation of mammary carcinoma cells. Reduced expression of decorin may facilitate cell growth, tumorigenesis and metastasis[9,19]. In human breast cancer tissues, decorin levels were decreased 2-5-fold when compared to normal breast tissue[14]. Treatment with decorin protein reduced primary tumor growth by 70% and eliminated observable metastasis in an orthotopic mammary carcinoma animal model injected with a metastatic breast cancer cell line. Adenoviral overexpression of decorin caused primary tumor retardation of 70%, in addition to greatly reducing the observation of metastasis [20]. The expression arrays revealed that decorin was down-regulated in tumor tissues, so we speculate that loss of decorin expression may contribute to the high proliferation of mammary epithelial cells. As a component of the ECM, decorin can bind several growth factors and their receptors, such as EGFR. After binding EGFR, decorin can inhibit cell proliferation by up-regulating the expression of p21.

EGFR on the cell surface is thought to play a pivotal role in cell proliferation, cell migration, and cell survival, but Marti et al.[21] also reported a nuclear distribution for EGFR, now called "nuclear EGFR," in primary adrenocortical carcinomas more than a decade ago. High levels of nuclear EGFR have subsequently been reported in many tumors, including those of the human breast, thyroid and cervix [22,23]. Thus two different signaling pathways, cytoplasmic/traditional and nuclear, have been identified. The cytoplasmic EGFR pathway often leads to tumorigenesis, tumor proliferation, metastasis, chemoresistance and radioresistance through the activation of Ras, PI-3K and STATs. The nuclear EGFR signaling pathway can escape the traditional transduction cascades and has different functions that depend on down-stream signaling molecules. Nuclear EGFR interacts with the DNA-binding transcription factors E2F1 and STAT3, and can accelerate G1/S cell cycle progression by up-regulating the expression of cyclin D1 and B-Myb. Cyclin D1 is a well-known oncogene whose overexpression is found in many cancers and is related to tumor progression and metastasis. Consistent with this mechanism, nuclear accumulation of EGFR is also associated with increased cell proliferation [22]. Increased expression of nuclear EGFR has been linked to poor clinical outcomes in patients with breast carcinoma [24] and oropharyngeal squamous cell carcinoma [25]. In the present study, we found that EGFR was located on the cell surface of mammary gland epithelial cells in five-month-old TA2 mice, while no nuclear EGFR was detected. In contrast, nuclear EGFR was detected in epithelial cells from normal mammary glands removed from spontaneous breast cancer-bearing TA2 mice as well as in breast cancer cells from those animals. In order to confirm the function of nuclear EGFR, we detected the expression of cyclin D1. A positive correlation between nuclear EGFR and cyclin D1 expression was observed both in mammary gland samples and breast cancer samples of cancer-bearing TA2 mice. The same result has also been observed in a cohort of breast carcinoma patients[24]. Our results suggest that nuclear translocation of EGFR may occur with increasing age, and that nuclear EGFR can promote the expression of cyclin D1, leading to a high proliferation index in mammary epithelial cells. Proliferating cell nuclear antigen (PCNA), the maestro of the replication fork, is a cofactor of DNA polymerases [26,27]. PCNA is now one of the most commonly used molecules to detect the proliferation index of tumor cells. Our results indicated that the mammary epithelial cells from cancer-bearing TA2 mice had a higher proliferation index (PCNA labeling index) than those of the five-month-old TA2 mice, and this was further confirmed by real-time PCR. In order to know whether nuclear EGFR could affect the expression of PCNA we also detected PCNA by immunohistochemical staining and real-time PCR. No correlation was found between PCNA and EGFR expression. Our results confirm that nuclear EGFR can indirectly up-regulate the expression of cyclin D1.

In the present study, expression profiles data showed that EGFR expression was down-regulated in cancer tissues compared with that of the matched mammary glands, in contrast to results previously reported for human breast cancer. In order to confirm our findings, we detected EGFR expression by real-time PCR and immunohistochemical staining. The results of real-time PCR and immunohistochemical staining were consistent with those of the gene arrays. As we know, EGFR is one of the prognostic factors and therapeutic targets for human breast cancers[22]. According to our results, EGFR may have different effect on the progression of breast cancer of TA2 mice and human beings. For TA2 mice, high level of EGFR played an important role in the carcinogenesis of its mammary gland epithelial cells, which needs further exploration.

## Conclusions

In briefly, our data suggest that the expression of decorin, EGFR and cyclin D1 in mammary epithelial cells changes with increasing age. Anestric mammary epithelial cells from five-month-old mice expressed low levels of EGFR. The kinase activity of this EGFR may have been attenuated in part by decorin. With increased age, decreased expression of decorin, increased expression of EGFR, especially nuclear EGFR, and increased expression of cyclin D1 may be the crucial alterations causing increased proliferation of mammary epithelial cells and these may partly conduce to the carcinogenesis of mammary gland epithelial cell of TA2 mice, which needs further studies.

## Abbreviations

ECM: extracellular matrix; EGFR: epithelial growth factor receptor; PCNA: proliferating cell nuclear antigen; Dcn: decorin; Igf2: insulin like growth factor 2; Mest: mesoderm specific transcript; Ndn: necdin; Peg3: paternally expressed 3; Bcl11a: B-cell CLL/lymphoma 11A; Cdh11: Cadherin 11; Fcgr2b: Fc receptor, IgG, low affinity IIb; Gpc3: Gypican 3; Vav3: Vav 3 oncogene; STAT3: signal transducer and activator of transcription; E2F1: E2F transcription factor 1; B-Myb: B-myeloblastosis oncogene

## Competing interests

The authors declare that they have no competing interests.

## Authors' contributions

GY carried out the animal experiment. ZS carried out pathologic examination. WQ carried out morphological observation. XS and CY carried out the immunohistochemical staining and counting. YZ performed the statistical analysis. ZX participated in the data analysis. SB carried out the design of the study and helped to draft the manuscript. All authors read and approved the final manuscript.
